# Choroidal Vascularity Index Assessment: A Potential Noninvasive Technique for Diagnosing Diabetic Nephropathy

**DOI:** 10.1155/2022/3124746

**Published:** 2022-02-24

**Authors:** Xue Han, Nan-Nan Du, Shuang Li, Zong-Li Diao, Li Fu, Wen-Hu Liu

**Affiliations:** ^1^Department of Nephrology, Beijing Friendship Hospital, Capital Medical University, Beijing, China; ^2^International Medical Center, Beijing Friendship Hospital, Capital Medical University, Beijing, China; ^3^Departmentof Ophthalmology, Beijing Friendship Hospital, Capital Medical University, Beijing, China

## Abstract

**Aims:**

This study aimed to compare the accuracy of the choroidal vascularity index (CVI) and diabetic retinopathy (DR) in the diagnosis of diabetic nephropathy (DN).

**Methods:**

We performed a cross-sectional study of 117 patients with proteinuria and diabetes mellitus (DM) in which 45 patients were diagnosed with DN by renal pathology. Demographic information, clinical features, and laboratory data were collected. A total of 234 eyes underwent evaluation of DR and the CVI using enhanced depth imaging-optical coherence tomography scans. We analyzed the association between the CVI and DN and compared the CVI and DR for diagnosing DN using area under receiver operating characteristic curves (AUROCs).

**Results:**

The severe nonproliferative DR and proliferative DR groups showed a lower CVI than the no DR and mild/moderate nonproliferative DR groups (*P* < 0.01 or *P* < 0.001). There was a significantly lower CVI in patients with DN stage III (63.01% ± 1.47%) compared with those in DN stages IIa (62.1% ± 1.41%, *P* < 0.001) and IIb (59.85% ± 1.98%, *P* < 0.01). The sensitivity and specificity of the CVI for diagnosing DN were 84% (71%–94%) and 95% (88%–99%), respectively, which were preferable to those of DR. The AUROCs for the CVI and DR for diagnosing DN were 0.932 and 0.831, respectively. The CVI outperformed DR for diagnosing DN (*P* < 0.05). The cutoff value of the CVI was 63.13%.

**Conclusion:**

The CVI might be a reliable noninvasive technique for predicting the pathological stage of DN and is superior to DR in diagnosing DN.

## 1. Introduction

Recent studies have suggested that the prevalence of diabetes mellitus (DM) is approximately 11% of the population worldwide, and DM affects 138 million adults in China [1, 2]. Diabetic nephropathy (DN) and diabetic retinopathy (DR) are common microvascular complications of DM [3]. In developed countries and developed regions of China, DN has become the primary cause of hemodialysis and kidney transplantation. Renal biopsy is the standard method for diagnosing DN, but its application is limited because of the invasiveness [3].

The renal and ocular microcirculations are analogous because the kidneys and eyes have similar physiological and pathological pathways. Therefore, the eyes are an accessible “window” through which we can speculate renal diseases including DN [4, 5]. DN is always accompanied by DR. DR is listed as one of the important clinical diagnostic criteria for DN according to the National Kidney Foundation/Kidney Disease Outcomes Quality Initiative (NKF/KDOQI) guidelines [6]. The choroid is the highly vascularized structure in the eye, from which the retina receives its blood supply [7]. Morphological abnormalities of the choroid may represent a systemic microvascular injury, and choroidal thickness has been reported to be associated with renal hemodynamics in essential hypertension [8]. The choroidal vascularity index (CVI) is defined as the ratio of the luminal area (LA) to the total choroidal area (TCA). The CVI is a novel tool for assessing the vascular network of the choroid using enhanced depth imaging-optical coherence tomography (EDI-OCT) scans [9–11]. The CVI is considered as a more stable and less variable parameter for estimating abnormalities in the choroidal vasculature than choroidal thickness [10]. Recent studies have shown that the CVI is significantly lower in patients with diabetes compared with those without diabetes and may be the main event of fundus oculi in diabetes, even when DR is absent [12–14].

The associations between choroidal vasculature and DN are yet to be systematically investigated. We make a tentative assumption that the CVI may have a better association with DN compared with DR. This study aimed to examine the association between DR and the CVI in patients with DN. Furthermore, we compared the diagnostic value of the CVI and DR for DN and aimed to determine if the CVI is more innovative and effective than DR.

## 2. Materials and Methods

### 2.1. Study Population

Research and ethics committee approval was obtained before the commencement of the study by the Bioethics Committee of Beijing Friendship Hospital, Capital Medical University (2018-P2-021-01). The study was implemented in compliance with the Declaration of Helsinki. Informed consent was obtained from all patients after explanation of the purpose, nature, and risk of all procedures. We performed a cross-sectional study between November 2018 and October 2020. A total of 125 patients with a history of proteinuria and DM who had percutaneous renal biopsy were screened in this study. All patients underwent fundus photography and EDI-OCT examination within 1 week of renal biopsy.

The inclusion criteria for the study were as follows: (i) age between 18 and 70 years; (ii) clinical history of proteinuria and DM for longer than 3 months; (iii) clinical indication for percutaneous renal biopsy; and (iv) written informed consent. The exclusion criteria for the study were as follows: (i) patients with end-stage renal disease (estimated glomerular filtration rate (eGFR) <15 ml/min/1.73 m^2^, kidney transplantation, or dialysis); (ii) patients with contraindications to percutaneous renal biopsy; (iii) patients with prior ocular history, including arterial or vein occlusions, uveitis, glaucoma, age-related macular degeneration, and any other retinal diseases; and (iv) patients with recent intraocular surgery and laser treatment within the last 3 months.

### 2.2. Clinical Examinations

Information on demographic characteristics, smoking status, history of diabetes, hypertension, and other chronic diseases was obtained. Hypertension was defined as systolic blood pressure ≥140 mmHg and diastolic blood pressure ≥90 mmHg. DM was defined as fasting plasma glucose levels ≥126 mg/dL (7.0 mmol/L), HbA1c values ≥6.5%, or 2 h plasma glucose levels ≥200 mg/dL (11.1 mmol/L) during an oral glucose tolerance test or in a patient with classic symptoms of hyperglycemia with random plasma glucose levels ≥200 mg/dL (11.1 mmol/L) [15].

Within 1 week of renal biopsy, fasting blood specimens were obtained for the measurement of hemoglobin, serum creatinine, serum albumin, and serum total cholesterol levels. A 24 h urine test was performed for the measurement of protein quantification. The eGFR was calculated using the Chronic Kidney Disease Epidemiology Collaboration equation as an evaluation of renal function [16].

### 2.3. Renal Pathology

Percutaneous renal biopsy was performed under ultrasound guidance. Renal tissue specimens were at least 1 cm in length and contained 10 intact glomeruli. Biopsy specimens were fixed in 10% formalin, embedded in paraffin, and stained with hematoxylin and eosin, Masson, periodic acid-silver methenamine, and periodic acid-Schiff (PAS). All specimens were subjected to immunofluorescence. According to the renal pathology, all of the patients were divided into two groups of patients with DN and non-DN patients. The pathological diagnostic standard for DN was proposed by the Research Committee of the Renal Pathology Society in 2010. DN was divided into four hierarchical glomerular lesions (classes I–IV) [17]. Discordant cases were reviewed by pathologists to reach a consensus.

### 2.4. DR Grading

The DR status obtained by fundus photochromy was analyzed in 234 eyes. According to the modified Early Treatment Diabetic Retinopathy Study, the DR grade was classified as no DR, mild nonproliferative diabetic retinopathy (NPDR), moderate NPDR, severe NPDR, and proliferative diabetic retinopathy (PDR) [18].

### 2.5. Image Binarization and CVI Calculation

**Figure 1:**
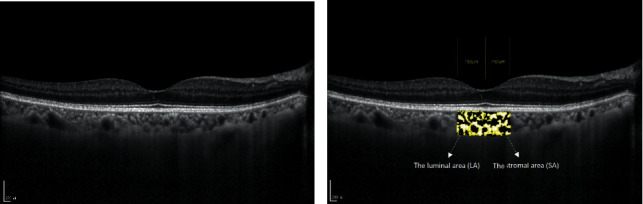
Representative images of processing to obtain the choroidal vascularity index using EDI-OCT scans. (a) Original OCT image. (b) Overlay image of an EDI-OCT scan with the region of interest obtained after image binarization. EDI-OCT: enhanced depth imaging-optical coherence tomography; DN: diabetic nephropathy; DR: diabetic retinopathy; PDR: proliferative diabetic retinopathy.

EDI-OCT scans were obtained in 234 eyes. After the EDI-OCT, the images were evaluated by three ophthalmologists. When two or more graders determined that the foveal choroid image was clearly identifiable, the image was considered acceptable and used for analysis. The OCT images were binarized and segmented using the protocol described by Sonoda et al. [19, 20]. The OCT image was opened in ImageJ software, and the polygon tool was used to select the TCA, which was added to the manager. The autolocal threshold was applied, with Niblack selected as the method. The color threshold tool was used to select the dark pixels, and this area was added to the region of interest manager. The TCA was defined as the selected area of the subfoveal choroid within a width of 1500 *μ*m (750 *μ*m on either side of the fovea), the LA was defined as the dark pixel area, the stromal area was defined as the light pixel area, and the CVI was defined as LA/TCA (Figure 1).

### 2.6. Statistical Analysis

Measurement data were expressed as mean ± standard deviation, and those that did not conform to a normal distribution were expressed as the median (interquartile range). Count data were expressed as values (%). The normality of data distribution was confirmed via the Kolmogorov–Smirnov test. The independent sample *t*-test (for normally distributed variables) and Mann–Whitney test (for nonnormally distributed variables) were used for the comparison of continuous variables between the two groups, while the chi-square test was used for the comparison of discrete variables. The logistic regression model was used for multivariate analysis. The diagnostic performance of DR and the CVI was assessed by receiver operating characteristic (ROC) curves. The areas under the ROC curves (AUROCs) were compared using the DeLong method. Sensitivity and specificity were calculated. The optimal cutoff value for the CVI was obtained using the criterion of maximal sum of sensitivity and specificity. Statistical analysis was performed using SPSS Statistics version 21 software and MedCalc software. *P* < 0.05 was considered statistically significant.

## 3. Results

### 3.1. Patients' Demographics and Clinical Characteristics

**Figure 2:**
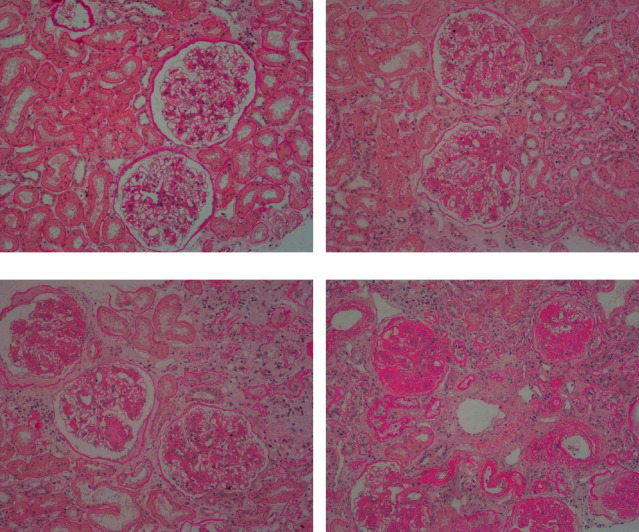
Representative images of renal pathology from patients with DN using the PAS stain (×200). (a) DN stage IIa: mild mesangial broadening. (b) DN stage IIb: severe mesangial broadening. (c) DN stage III: nodular glomerulosclerosis (Kimmelstiel–Wilson nodules). (d) DN stage IV: advanced glomerulosclerosis. DN: diabetic nephropathy; PAS: periodic acid-Schiff.

From November 2018 to October 2020, 125 patients who fulfilled the study criteria were enrolled. Eight patients were excluded because of unreliable ophthalmologic images. Therefore, 117 patients and 234 eyes were eligible for statistical analysis. A total of 45 cases of DN were diagnosed by renal pathology. There were 72 cases of non-DN, including 15 cases of mesangial proliferative glomerulonephritis, 34 cases of membranous nephropathy, 12 cases of minimal change disease, 1 case of membranoproliferative glomerulonephritis, and 10 cases of other causes. The patients' characteristics are shown in Table 1. Different stages of renal pathology in patients with DN are shown in Figure 2.

### 3.2. Predictive Factors of DN

By univariate analysis, a history of DM, a history of smoking, DR, and the CVI were significantly associated with DN (all *P* < 0.05). All other factors, including age, sex, a history of hypertension, eGFR, hemoglobin, serum creatinine, serum albumin, serum total cholesterol, and 24 h urine protein, were not significantly associated with DN. The DN group had a longer history of diabetes and had a higher incidence of a history of smoking and DR compared with the non-DN group (all *P* < 0.05). The CVI was significantly lower in the DN group (61.54 ± 2.14) compared with the non-DN group (65.09 ± 1.35, *P* < 0.001) (Table 1). By logistic regression analysis, a history of DM (≥10 years) (odds ratio = 39.18, *P*=0.001), DR (odds ratio = 8.89, *P* < 0.05), and a decreased CVI (odds ratio = 0.23, *P* < 0.001) were independent predictors of DN (Table 2).

### 3.3. Association between DR and the CVI

**Figure 3:**
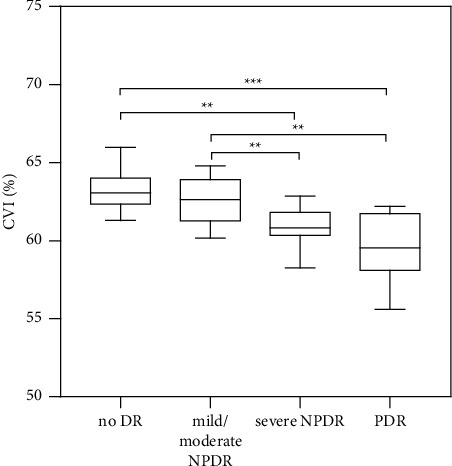
The CVI in different DR groups in 90 eyes of 45 patients with DN. CVI: choroidal vascularity index; DR: diabetic retinopathy; NPDR: nonproliferative diabetic retinopathy; PDR: proliferative diabetic retinopathy; DN: diabetic nephropathy. ^*∗∗*^*p* < 0.01 and ^*∗∗∗*^*p* < 0.001.

We investigated 90 eyes in 45 patients with DN, including 20 no DR, 24 mild/moderate NPDR, 20 severe NPDR, and 26 PDR eyes. The mean CVIs in each group were 63.27% ± 1.42%, 62.56% ± 1.51%, 60.95% ± 1.29%, and 59.73% ± 2.13%, respectively. Notably, the severe NPDR group showed a significantly lower CVI compared with the no DR (*P* < 0.01) and mild/moderate NPDR groups (*P* < 0.01). Similarly, the PDR group showed a significantly lower CVI compared with the no DR (*P* < 0.001) and mild/moderate NPDR groups (*P* < 0.01). There were clinical differences in the CVI between the no DR and mild/moderate NPDR groups and between the severe NPDR and PDR groups, but these differences were not significant (Figure 3).

### 3.4. Association between DN and the CVI

**Figure 4:**
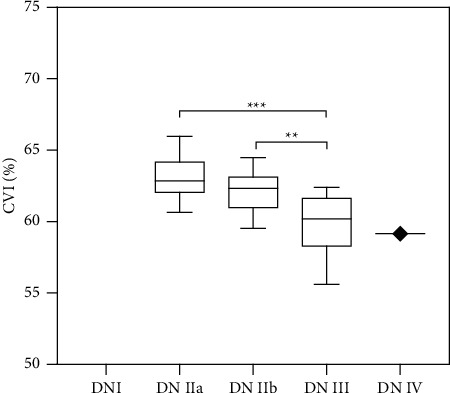
The CVI in different pathological stages of 45 patients with DN. CVI: choroidal vascularity index; DN: diabetic nephropathy.^*∗∗*^*p* < 0.01 and ^*∗∗∗*^*p* < 0.001.

We investigated 45 patients with DN, including 15 with DN-IIa, 13 with DN-IIb, 16 with DN-III, and 1 with DN-IV. The mean CVIs in each group were 63.01% ± 1.47%, 62.1% ± 1.41%, 59.85% ± 1.98%, and 59.16%, respectively. Notably, the DN-III group showed a significantly lower CVI compared with the DN-IIa (*P* < 0.001) and DN-IIb groups (*P* < 0.01) (Figure 4). Additionally, we found no significant association between the CVI and eGFR in patients with DN (data not shown).

### 3.5. Diagnostic Accuracy of DR and the CVI in DN

**Figure 5:**
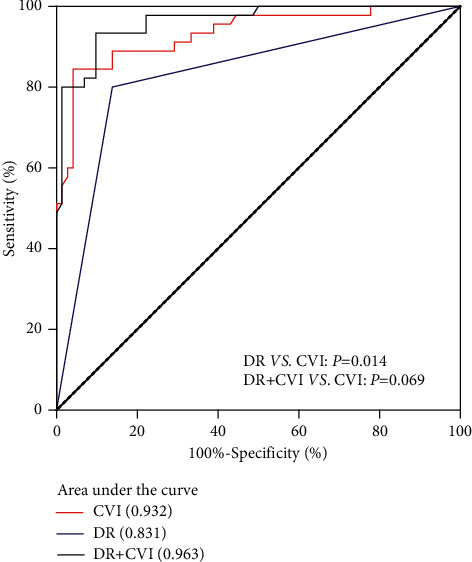
Receiver operating characteristic curves (ROCs) of the CVI, DR, and the CVI combined with DR to identify participants with DN. CVI: choroidal vascularity index; DR: diabetic retinopathy; DN: diabetic nephropathy.

The diagnostic accuracy of DR and the CVI in DN was compared using a paired ROC curve. AUROCs of DR and the CVI were 0.831 (95% confidence interval (CI): 0.748–0.913) and 0.932 (95% CI: 0.871–0.971), respectively. The difference between the AUROCs of the CVI and DR was 0.101 (*P* < 0.05) (Figure 5). The sensitivity of DR was 0.80 (95% CI: 0.65–0.90), and specificity was 0.86 (95% CI: 0.76–0.93). The sensitivity of the CVI was 0.84 (95%CI: 0.71–0.94), and specificity was 0.95 (95% CI: 0.88–0.99). The sensitivity and specificity of the CVI were higher than those of DR (*P* < 0.05). The optimal cutoff value to maximize the sum of sensitivity and specificity was 63.13% for the CVI (Table 3).

Additionally, we combined DR and the CVI as a model and evaluated the diagnostic value of this model for DN. The sensitivity and specificity of the combined index to predict DN were 0.93 (95% CI: 0.82–0.99) and 0.90 (95% CI: 0.81–0.96), respectively. The AUROC of DR combined with the CVI was 0.963 (95% CI: 0.932–0.994), but there was no difference between the AUROCs of the combined index and the CVI (*P* > 0.05) (Figure 5 and Table 3).

## 4. Discussion

Renal microvascular changes, such as renal arteriolosclerosis, interstitial ischemia, and the loss of the peritubular capillaries, are considered as typical pathological features of DN [21]. Renal microvascular abnormalities can be reliably evaluated by a renal biopsy. However, the invasive operation and limitations of renal biopsy mean that monitoring the progression of DN and responses to treatment is impossible. Therefore, a noninvasive method for diagnosing DN is required. Because the renal and retinal circulations share similar physiological and pathological characteristics, the existence of DR often predicts the occurrence of DN. The choroid is a large vascular organ, which can fully reflect microvascular damage and loss [7]. Whether the CVI is a better indicator of DN compared with DR needs to be further investigated.

This study analyzed the associations between variables and the occurrence of DN by univariate analysis. We found that patients in the DN group had a longer history of DM, a higher incidence of smoking and DR, and a lower CVI compared with those in the non-DN group. Logistic regression analysis was used to further evaluate the predictive factors of DN, and it showed that a history of DM (≥10 years), a lower CVI, and DR were associated with DN. Proteinuria is also considered as another important clinical diagnostic indicator for DN according to the 2007 NKF/KDOQI guidelines [6]. Our study showed that there was no significant difference in albuminuria between patients with and those without DN, which can be explained by the composition of diseases in the non-DN group. In the non-DN group, glomerulonephritis and nephrotic syndrome were the main disease types, which were characterized by proteinuria and even a large amount of proteinuria.

Because a decline in the CVI can potentially predict the occurrence of DN, we further investigated whether the CVI is related to DN stages. According to the renal pathology, 45 patients were divided into DN stages IIa–IV. We found that the eyes of patients in DN stage III showed a reduced CVI compared with those in DN stages IIa and IIb. These findings suggest that, with increasing severity of renal pathology, the CVI becomes lower as microvascular lesions in the eyes become more severe. The absence of DN stage I was due to mild clinical manifestations in the patients, and renal puncture was not necessary for patients with no proteinuria or only microproteinuria. The small number of patients with DN stage IV can be explained by glomerulosclerosis and poor renal function, which led to a lost opportunity for renal biopsy. However, we found no significant association between the CVI and eGFR in patients with DN. This is because there are too many factors that affect the eGFR, including age, the course of DN, quantity of proteinuria, blood pressure, medication, and other chronic diseases.

Tan et al. showed that 38 eyes in patients with DM had a lower CVI compared with controls, but they did not investigate the CVI according to the DR stage [12]. In another study, the eyes in patients with DM showed a lower CVI than those of healthy controls. The PDR group showed a lower CVI than the healthy control, no DR, and mild/moderate NPDR groups [13]. In the current study, we determined the CVI in patients with DN. Notably, the PDR and severe NPDR groups showed a lower CVI than the no DR and mild/moderate NPDR groups. Therefore, the CVI decreased as DR progressed. In this study, we did not include a healthy control group because we found that the CVI in the healthy control group was 66.84% ± 1.45% in our previous study [22]. In the current study, the mean CVI was 63.27% ± 1.42% in patients with DN without DR, which is a lower value than that in healthy controls.

The choroid is one of the most vascularized tissues in humans, and alterations of the choroidal function are associated with the pathogenesis of ocular and systemic diseases [23]. Choroidal blood flow accounts for 90% of ocular blood flow. Compared with the retina, the choroid is more sensitive to the ocular microcirculation disorder in the presence of diabetes. Some studies demonstrated that the decreased blood flow of the choroid could be involved in diabetic eyes before the onset of DR [24–26]. So, we speculate that the CVI may have a better association with DN compared with DR. To compare the diagnostic value of DR and the CVI for DN, we compared the AUROCs of DR, the CVI, and DR combined with the CVI. Our study showed that the sensitivity and specificity of the CVI for diagnosing DN were preferable to those of DR. The cutoff value of the CVI was 63.13%, which means a CVI lower than 63.13% may indicate the possible onset of DN, especially when patients have other clinical manifestations of DN, such as a long history of diabetes and proteinuria. However, there was no difference between the AUROCs of the combined index and the CVI. This suggests that it may be possible to use CVI instead of DR as the routine examination index for diabetic patients, especially with kidney injury. Of course, the lack of a statistical difference of this may also be due to the small sample size.

This study has some limitations. The number of patients with DN was not large enough for definitive conclusions. This is mainly due to the method of DN being diagnosed. Unlike other glomerular diseases, DN can be diagnosed only by clinical indicators independent of renal pathology, although renal pathology is the gold standard. The sample size needs to be further increased, and external validation on the results of this study needs to be conducted.

In conclusion, we found that assessment of the choroidal vascular network using the CVI might be a useful indicator for monitoring pathological damage in patients with DN even without DR. The CVI is a new noninvasive tool that potentially makes evaluation and diagnosis of DN more accurate than using DR. Further clinical studies with a larger sample size should be carried out to better understand the diagnostic potential of CVI.

## Figures and Tables

**Table 1:** Characteristics of the study population of participants with and without DN.

Characteristics	Overall (*n* = 117, eyes = 234)	DN patients (*n* = 45, eyes = 90)	Non-DN patients (*n* = 72, eyes = 144)	*P* value
Age, y	56.3 ± 11.7	54.2 ± 10.5	57.6 ± 12.3	0.123
Males, %	67.5	75.6	62.5	0.142
DM, y	9.1 ± 7.6	13.5 ± 7.5	5.6 ± 5.3	＜0.001
Hypertension, %	72.6	73.3	72.2	0.896
Smoking, %	54.7	66.7	47.2	0.04
Albumin, g/L	32.0 ± 7.4	32.9 ± 6.2	31.1 ± 8.1	0.194
Hemoglobin, g/L	123 ± 21	119.8 ± 22.0	125.4 ± 21.5	0.180
Total cholesterol, mmol/L	5.08 ± 1.53	4.45 ± 1.16	5.47 ± 1.51	0.098
24-hour urine, g	2.89 ± 2.39	2.66 ± 2.16	3.03 ± 2.54	0.424
Creatinine, *μ*mol/L	115.5 ± 81.4	122.9 ± 98.4	104.3 ± 68.4	0.230
eGFR, ml/min/1.73㎡	74.21 ± 30.69	70.08 ± 28.77	76.79 ± 31.76	0.251
DR, %	37.6	86.1	22.2	＜0.001
CVI	63.72% ± 2.42%	61.54% ± 2.14%	65.09% ± 1.35%	＜0.001

Continuous variables are presented as the mean ± SD; categorical data are presented as values (%). DN: diabetic nephropathy; DM: diabetes mellitus; eGFR: estimated glomerular filtration rate; DR: diabetic retinopathy; CVI: choroidal vascularity index.

**Table 2:** Logistic regression of risk factors for DN.

Variable	Classification	OR (95% CI)	*P* value
DM	≥10 years	39.18 (4.50–341.28)	0.001
Smoking	Yes	8.81 (1.02–64.40)	0.051
DR	Yes	8.89 (1.53–51.77)	0.015
CVI	Every increase of 1%	0.23 (0.11–0.46)	＜0.001

The logistic regression model was used for multivariate analysis. DN: diabetic nephropathy; DM: diabetes mellitus; DR: diabetic retinopathy; CVI: choroidal vascularity index; OR: odds ratio.

**Table 3:** Diagnostic value of the CVI and DR in DN.

Variable	AUROCs (95% CI)	Sensitivity (95% CI)	Specificity (95% CI)	Cutoff (MS)
DR	0.831 (0.748–0.913)	0.80 (0.65–0.90)	0.86 (0.76–0.93)	
CVI	0.932 (0.871–0.971)	0.84 (0.71–0.94)	0.95 (0.88–0.99)	63.13
DR + CVI	0.963 (0.932–0.994)	0.93 (0.82–0.99)	0.90 (0.81–0.96)	

The diagnostic performance of DR and the CVI was assessed by ROC curves. The AUROCs were compared using the DeLong method. Sensitivity and specificity were calculated. The optimal cutoff value for the CVI was obtained using the criterion of maximal sum of sensitivity and specificity. CVI: choroidal vascularity index; DR: diabetic retinopathy; DN: diabetic nephropathy; ROC: receiver operating characteristic; AUROCs: the areas under the ROC curves; MS: maximal sum of sensitivity and specificity.

## Data Availability

The data are available upon request to the corresponding author.
